# Criteria ruling particle agglomeration

**DOI:** 10.3762/bjnano.12.81

**Published:** 2021-09-29

**Authors:** Dieter Vollath

**Affiliations:** 1 NanoConsulting, Primelweg 3, 76297 Stutensee, Germany

**Keywords:** agglomeration, enthalpy, entropy, simulation, surface energy, van der Waals interaction

## Abstract

Most of the technically important properties of nanomaterials, such as superparamagnetism or luminescence, depend on the particle size. During synthesis and handling of nanoparticles, agglomeration may occur. Agglomeration of nanoparticles may be controlled by different mechanisms. During synthesis one observes agglomeration controlled by the geometry and electrical charges of the particles. Additionally, one may find agglomeration controlled by thermodynamic interaction of the particles in the direction of a minimum of the free enthalpy. In this context, one may observe mechanisms leading to a reduction of the surface energy or controlled by the van der Waals interaction. Additionally, the ensemble may arrange in the direction of a maximum of the entropy. Simulations based on Monte Carlo methods teach that, in case of any energetic interaction of the particles, the influence of the entropy is minor or even negligible. Complementary to the simulations, the extremum of the entropy was determined using the Lagrange method. Both approaches yielded identical result for the particle size distribution of an agglomerated ensemble, that is, an exponential function characterized by two parameters. In this context, it is important to realize that one has to take care of fluctuations of the entropy.

## Introduction

Many properties of technical importance of nanomaterials depend on the particle size. Typical examples are superparamagnetism, luminescence, or energetic applications. In many cases, the particles show in an “as produced” state the requested size distributions. However, during handling or storage of these materials, the particle size increases due to agglomeration. This leads to a product of reduced applicability.

Therefore, concerning the formation and handling of nanoparticles, agglomeration is the crucial problem. Detailed considerations lead to a few fundamentally different mechanisms. Synthesis of larger particles may be carried out in the gas phase, where the probability of collision is controlled by the geometry of the particles or forces among the particles [1]. Alternatively, in case of existing particles, the agglomeration is controlled by the minimum of free enthalpy. In this case, one has to distinguish two different aspects. Either the particles form agglomerates without exchanging energy, such a system is described by the maximum of the entropy [2–3], or agglomeration is connected to energetic interaction, for example, particle sintering, leading to a reduction of the surface-related enthalpy. In this case, the minimum of free enthalpy rules the agglomeration. The cases described above are essentially different to the one observed when an ensemble of particles stored in a vessel forms clusters [4].

## Basic Considerations

Generally, in the description of thermodynamic systems, the Jaynes principle of maximum entropy [5–8] is used as a criterion. However, this principle is a necessary condition but not a sufficient one [3,9]. Since this principle is insufficient, for example, in the case of energetically interacting particles, one has to apply the Gibbs principle of a minimum of free enthalpy. This means that one has to introduce additional criteria for a complete description of the system. The principle of maximum entropy is based on either the Boltzmann entropy or the Gibbs entropy of mixing. In the description of the thermodynamics of sufficiently large ensembles of particles, these two descriptions are identical.

The Boltzmann entropy *S* is given by *S* = *k*·ln(*W*), with *k* the Boltzmann constant. For an ensemble consisting of *N* elements subdivided in *I* groups, each one consisting of *n_i_* elements, the number of possibilities *W* is described as:

[1] $$W=\frac{N!}{\prod_{i}n_{i}},\text{ constraint }N=\sum_{i}n_{i}.$$

In the further discussions, for simplicity, instead of the entropy *S*, a reduced entropy S* = *S*/*k* is used. After applying Stirling’s equation, for large numbers of particles, the Boltzmann entropy may be rewritten as:

[2] $$S^{*}=\ln\left(W\right)=N\ln\left(N\right)-N-\sum_{i}n_{i}\ln\left(n_{i}\right)+\sum_{i}n_{i}.$$

Setting *n_i_*/*N* = *p_i_*, one obtains the Gibbs entropy of mixing:

[3] $$\frac{S^{*}}{N}=-\sum_{i}\frac{n_{i}}{N}\ln\left(\frac{n_{i}}{N}\right)=-\sum_{i}p_{i}\ln\left(p_{i}\right).$$

Equation 2 and Equation 3 demonstrate that, in this special case, the two definitions of the entropy above are equivalent. In this context, it is important to mention that during agglomeration, which is a dynamic process, the number of objects is not constant. At the beginning of the agglomeration process, the number of initial particles is *N*_tot_. During agglomeration, the number of objects *N* in the system is reduced to *N* < *N*_tot_. At each step of agglomeration the number of independent particles in the system is 
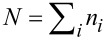
. This number is smaller than the original number of initial particles, 
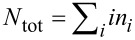
.

To apply this system to a problem, for example, the agglomeration of particles, one has to solve a system with *I* + 1 side conditions. A solution is possible by applying the Lagrange formalism. This may be difficult as it would be necessary to find solutions for a non-linear system of equations with *I* + 1 unknown parameters.

As mentioned above, the Jaynes principle is a necessary but not a sufficient condition; therefore, an additional constraint must be introduced. It is possible, in many cases necessary, to use the total energy *E*_tot_ as constraint in connection with other parameters describing the equilibrium of the system [9]. An appropriate selection of side conditions is necessary to obtain reliable results. Looking at these limitations in context of numerical solutions, it is advised to search for better approaches for the determination of the distribution function of the probabilities *p_i_*. In other words, the actual question is: Is there an inherent valid distribution function of sizes of the agglomerates?

A maximum of the entropy is a necessary but not a sufficient condition for an equilibrium. Therefore, it is appropriate to determine the change of the free enthalpy Δ*G* caused by agglomeration, as:

[4] $$\Delta G=\Delta H-TS=\sum_{i}\Delta h_{i}^{*}+kTN\sum_{i}p_{i}\ln\left(p_{i}\right).$$

In Equation 4, *T* is the temperature, Δ*G* is the change of the free enthalpy and Δ*H* is the change of the enthalpy. It is obvious that the change of the binding energy, 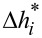, may depend on the number of particles in an agglomerate. Since the binding energy depends on the actual arrangement of the particles in the cluster, one has to assume a new distribution function for each possible arrangement [10]. However, generally, this arrangement is unknown. Furthermore, this term contains the change of the surface energy between the starting arrangement and the agglomerated state. Looking at the agglomeration of nanoparticles, the binding energy of two particles is relatively small. One of the possibilities for the exchange of energy between particles could be the van der Waals interaction. However, for particles touching each other, the van der Waals energy is not properly defined [11]. Since the real arrangement of the particles in a cluster is not known, as a first approximation, the energy of interaction was assumed to be proportional to the number of contact points. As smallest number, for each particle one contact point was assumed. Luo et al. [12] estimate the energy of interaction in the range of approximately *h*_0_ = 10 eV (1.6 × 10^−18^ J). This is a relatively large value in comparison to older data. According to Vold [13], this energy should be larger than 10*kT*. As first approach, the enthalpy of interaction was approximated by Δ*h_i_* = *h*_0_(*i* − 1). This means that for each particle connected to an agglomerate only one binding is assumed. However, in many cases, the interaction based on the surface energy rules the process of agglomeration. Most important, in this context, is the change of the surface energy due to the alteration of the surface as the relative surface decreases with increasing particle size. Assuming the surface-related enthalpy *h*_1_ = πσ*v*_1_^2/3^, with σ being the surface energy of a particle with the smallest volume *v*_1_, an agglomerated particle consisting of *n* particles has the reduced surface energy:

[5] $$\Delta h_{i}=\pi \sigma {\left(iv_{1}\right)}^{2/3}-\pi \sigma v_{1}{}^{2/3}.$$

Equation 5 does not take into account that the surface energy may depend on the particle size [14]. Equation 4 is now modified to:

[6] $$\Delta G=\sum_{i}\pi \sigma \left[{\left(iv_{1}\right)}^{2/3}-v_{1}{}^{2/3}\right]+kTN\sum_{i}p_{i}\ln\left(p_{i}\right).$$

The concept outlined above is based on a quasi-static process. However, each actual process is, to some extent, superimposed by fluctuations. According to Mishin [15] this behavior is also valid for the entropy in an equilibrium state. Lastly, this fact is not in accordance with the “Second Law of Thermodynamics”, which does not allow a reduction of the entropy to occur. Therefore, Mishin [15] had to put this into a broader theoretical framework. One of the fundamental rules for the entropy in a fluctuating system requires to distinguish between an average level and a maximum level, which must not be exceeded. It is necessary to distinguish between the entropy fluctuation phenomenon described by Mishin and that explained earlier by Crooks [16] for small systems.

## Results

### Results of the entropy simulation

**Figure 1:**
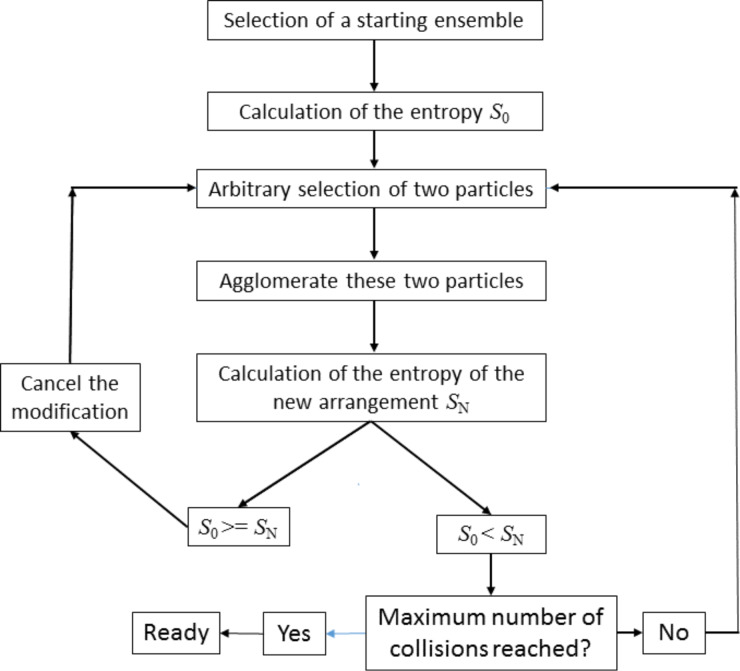
Flow chart of the algorithm to calculate the agglomeration of an ensemble in the direction of a maximum entropy.

As described in the previous chapter, the maximum entropy principle gives clear indications about the rules governing ensembles of particles. However, the application is difficult because of the necessary side conditions. The situation may be improved if more a priori information exists about the distribution leading to the maximum of the entropy. The formation of agglomerates is a random process driven in the direction of a maximum of the entropy. Such a process can be simulated numerically using Monte Carlo calculations. The basic idea for such an algorithm consists of the following steps: As basic assumption, an ensemble of *N*_tot_ equal particles is selected. As these particles shall be distinguishable, a number is given to each of these particles. At each step, a random number generator selects two particles. In a first step, these particles are agglomerated in such a way that the entropy of the resulting distribution is larger than the previous one. After each successful step, one reduces the number of elements, particles plus agglomerates, in the distribution by one. This process is repeated. The corresponding algorithm is sketched in Figure 1.

To obtain statistically significant results, the calculations were performed with 10^5^ and 10^7^ particles in the starting configuration. In the range between 0.3*N*_tot_ and 0.35*N*_tot_ iteration steps, which is equivalent to collisions between particles, the calculation was stopped, because each additional collision led to a configuration with reduced entropy. Consequently, the side condition that allows only collisions leading to an increase of the entropy had to be removed. Obviously, because of the fluctuations, an algorithm as explained above does not allow for a determination of the maximum level of the entropy.

**Figure 2:**
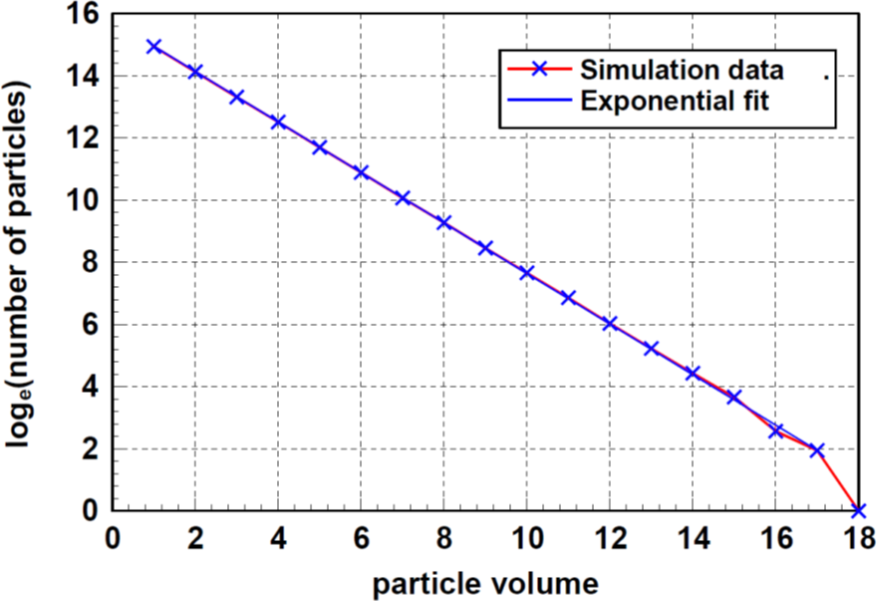
Plot of the distribution function after 4.7 × 10^6^ collisions of a starting ensemble consisting of 10^7^ particles, the ordinate is transformed to the natural logarithm, log*_e_*(*N_i_*) = log*_e_*(*a*) − *bi*. Additionally, the fit of this distribution with an exponential function is depicted. One realizes a perfect fit for statistically significant numbers of particles.

As result of the calculations, one obtains a correlation between the number of collisions and the resulting particle size distribution. Taking a fixed value of collisions one can display the probability distribution for the particle sizes. An example is shown in Figure 2. This distribution is displayed on a logarithmic scale for 4.7 × 10^6^ collisions for a starting ensemble consisting of 10^7^ particles. Additionally, the approximation with an exponential function *N_i_* = *a*·exp(−*bi*), where *a* and *b* are parameters describing the distribution function, is depicted. The variable *i* stands for the volume of the particles in multiples of the initial particle size. Except for particle numbers of less than 20 one realizes a perfect approximation. However, in the latter case, the particle numbers are no longer statistically significant.

**Figure 3:**
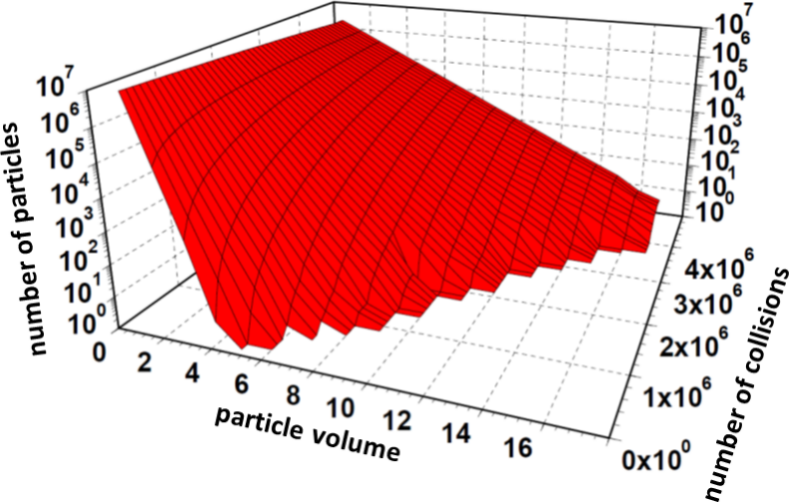
Number of particles as function of the particle volume and the number of collisions. This simulation was started with 10^7^ primary particles. The calculations were performed up to 4.7 × 10^6^ collisions. For each number of collisions, the distribution function follows obviously an exponential function.

Figure 3 shows the number of particles as function of the particle volume and the number of collisions in the range between 10^5^ and 4.5 × 10^6^. One can observe that between the logarithm of the number of particles and the particle volume is a linear correlation, regardless of the number of collisions. As it is visible in Figure 3, the parameter *b*, describing the inclination, decreases with increasing number of collisions.

**Figure 4:**
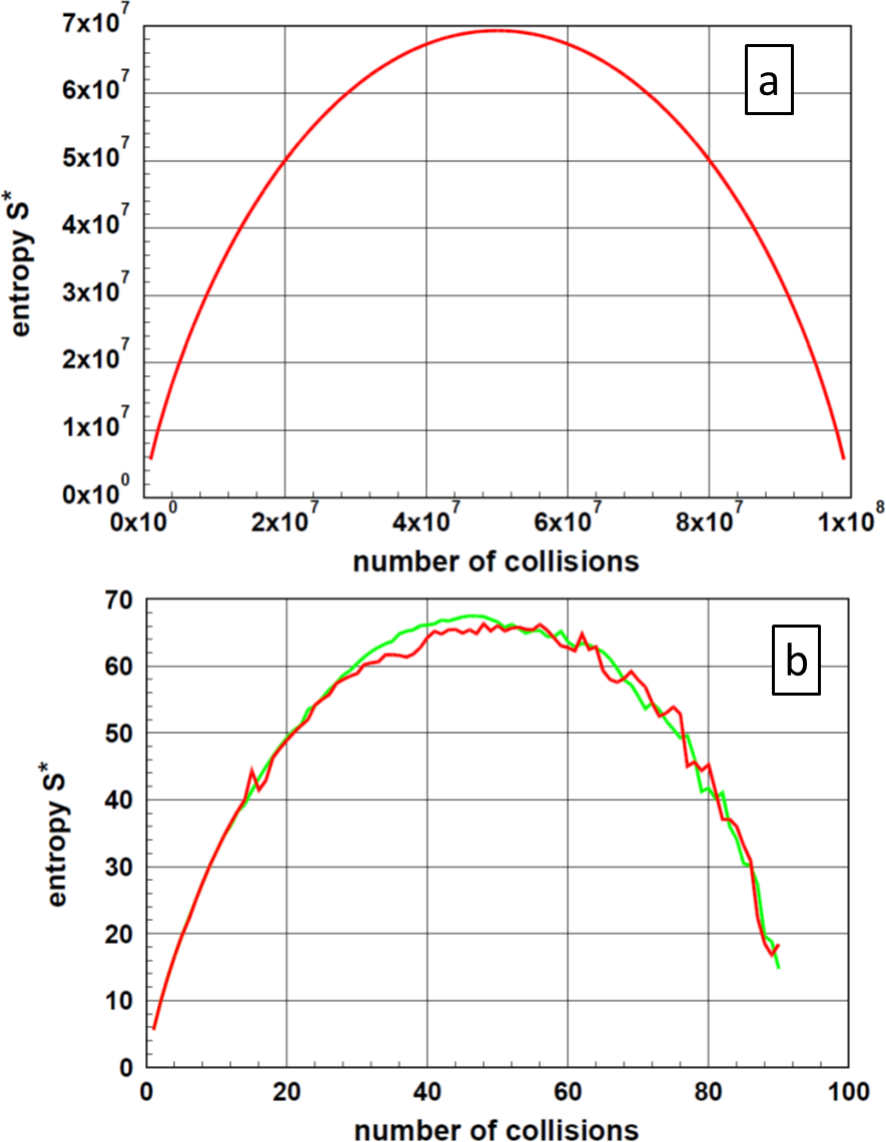
Entropy S* as function of the number of collisions. For this example, the starting ensemble consisted of 10^8^ particles (Figure 4a). To demonstrate the fluctuations of the entropy, an additional graph of the entropy S* for two ensembles consisting of 10^2^ particles is depicted (Figure 4b). In the latter case, one realizes some scatter of the entropy values. In an experiment, only fluctuations are observable after the system has reached the maximum.

The determination of the entropy S* using Equation 3 leads, after a steep increase at low numbers of collisions to a maximum at *N*_tot_/2, followed by a steep decrease. These relations are depicted in Figure 4 for an ensemble of *N*_tot_ = 10^8^ particles. The graphs show a maximum in the vicinity of *N*_tot_/2. Because of the unavoidable fluctuations, simulations cannot determine the exact position of the maximum. In case of an experimental realization, one must be aware that the progress of agglomeration stops at the maximum of the entropy. After reaching this point, one observes only fluctuations. To demonstrate entropy fluctuations, Figure 4a displays the entropy S* for an ensemble of 10^8^ particles, while Figure 4b displays the same situation for a small ensemble consisting of 10^2^ particles. A comparison of Figure 4a and Figure 4b demonstrated that only the absolute value of the entropy depends on the number of particles in the ensemble. However, the shape of the function of entropy versus number of collisions does not depend on the size of the ensemble.

Starting with the finding that the probability distribution of particle sizes follows an exponential function, it is possible to calculate the parameters for the maximum of the entropy in detail. Assuming an ensemble consisting of *N*_tot_ particles, which has undergone *n*_coll_ collisions, the number of particles with the size *i* is formulated as:

[7] $$N_{i}=\left(N_{\text{tot}}-n_{\text{coll}}\right)\exp\left(-ib\right).$$

Therefore, the probability *p_i_* for these particles follows as:

[8] $$p_{i}=\frac{N_{i}}{\left(N_{\text{tot}}-n_{\text{coll}}\right)}=\exp\left(-ib\right).$$

and, consequently, the entropy S* as:

[9]
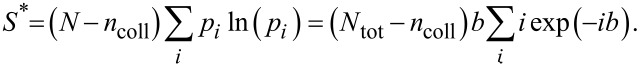


To calculate the values of *b* and *n*_coll_, where the entropy is extremal, one has to take into account the constraint

[10] $$N_{\text{tot}}=\sum_{i}iN_{i}\Rightarrow N_{\text{tot}}-\left(N_{\text{tot}}-n_{\text{coll}}\right)\sum_{i}i\exp\left(-ib\right)=0.$$

Using Equation 9 and Equation 10, the extremum of the Lagrange function


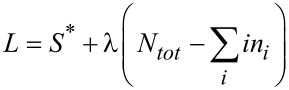


allows for calculating the unknown parameters as

[11] $$\begin{array}{c} \frac{\partial L}{\partial n_{\text{coll}}}=-\left(b+\lambda \right)\sum_{i}i\exp\left(-ib\right)=0\;\Rightarrow \;\lambda =-b,\\ \frac{\partial L}{\partial b}=\sum_{i}i\exp\left(-ib\right)+\left(\lambda -b\right)\sum_{i}i^{2}\exp\left(-ib\right)=E_{1}=0,\\ \frac{\partial L}{\partial \lambda }=\left[N_{\text{ges}}-\left(N_{\text{ges}}-n_{\text{coll}}\right)\sum_{i}i\exp\left(-ib\right)\right]=E_{2}=0. \end{array}$$

The system of nonlinear equations is reduced to

[12] $$F=\left|E_{1}\right|+\left|E_{2}\right|=\min$$

and solved using a program for nonlinear regression analysis [17]. The results of these numerical calculations, together with the evaluation of the simulation calculations are given in Table 1. To improve readability, in this table, instead of *n*_coll_ the parameter *a* = *n*_coll_/*N*_tot_ is given. This table compares the results obtained from Equation 11 and from simulations. As fluctuation processes influence the simulation, there is a difference between the calculated values according to Equation 12 and those obtained from simulation. In the case of 10^5^ particles, two values from different runs for the parameter *b* are given.

**Table 1:** Comparison of the parameters *a* and *b* in 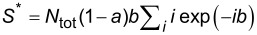 calculated from Equation 12 and simulation for the maximum of the entropy. As the results from simulation are influenced by thermal fluctuations, for *N*_tot_ = 10^5^ the results of two different runs are given.

*N*_tot_	Parameter	Calculated solution (Equation 11)	Simulation

10^8^	*a*	0.50391015	0.5
	*b*	0.695748181	0.690063
10^5^	*a*	0.501452321	0.5
	*b*	0.694029101	0.698, 0.693

The data displayed in Table 1 show, except for minor deviations caused by thermal fluctuations in the simulation, a perfect agreement between the results of these two approaches. Several calculations using Equation 12 showed, if at all, only a negligible dependency of the parameters *a* and *b* on the total number of particles in the system.

**Figure 5:**
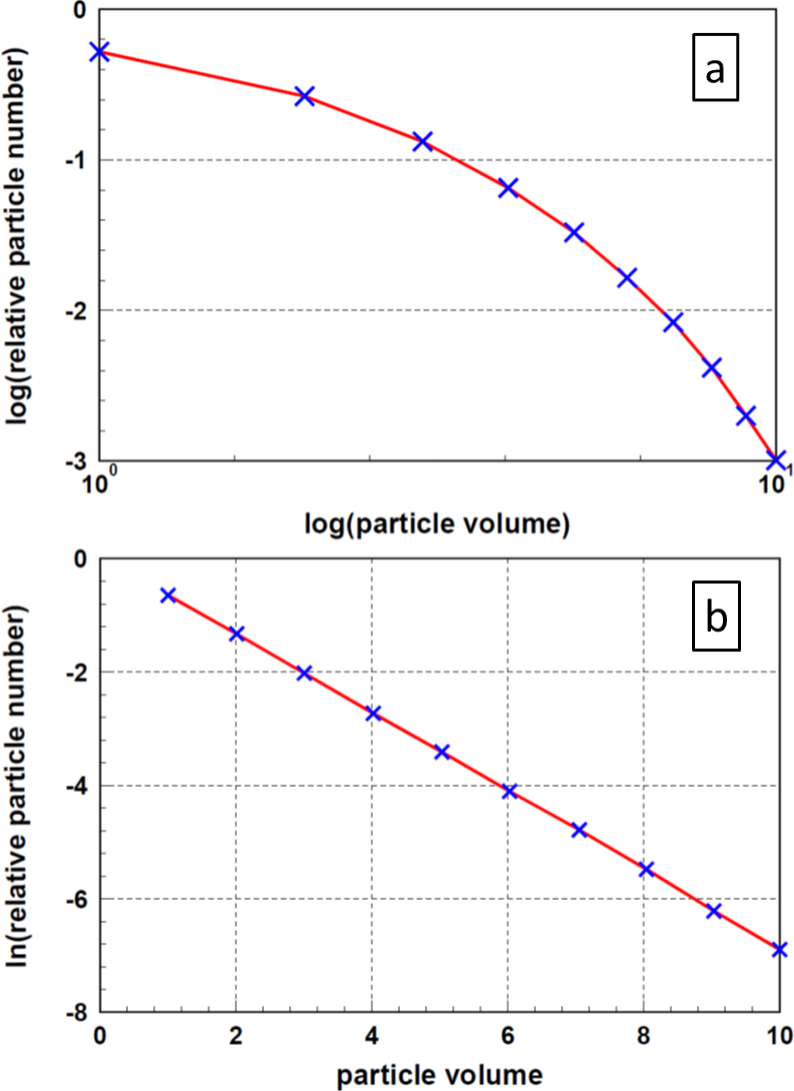
Distribution function of particle sizes according to Kätelhön and co-workers [18]. Figure 5a displays the results as published by the authors in a double logarithmic plot (base 10), Figure 5b shows the same results, in accordance with Figure 2, in a linear–logarithmic (base *e*) plot.

Using a different mathematical approach to determine the distribution function and the minimum of the Lagrange function, Kätelhön et al. [18] found the results displayed in Figure 5. In this case, the distribution function was calculated pointwise. Figure 5 displays these results in two different ways. Figure 5a shows these results as published by the authors on a double logarithmic scale using decimal logarithms. These data are replotted in Figure 5b, similar to Figure 2. One recognizes a perfect straight line; the parameter *b* determined from this plot is 0.6929. This value coincides, within the scattering ranges caused by fluctuations, exactly with the values in Table 1. This fact confirms the correctness of the procedure reported in the previous sections. Furthermore, the selection of an exponential function for the particle size distribution is justified.

### Results of enthalpy simulations

**Figure 6:**
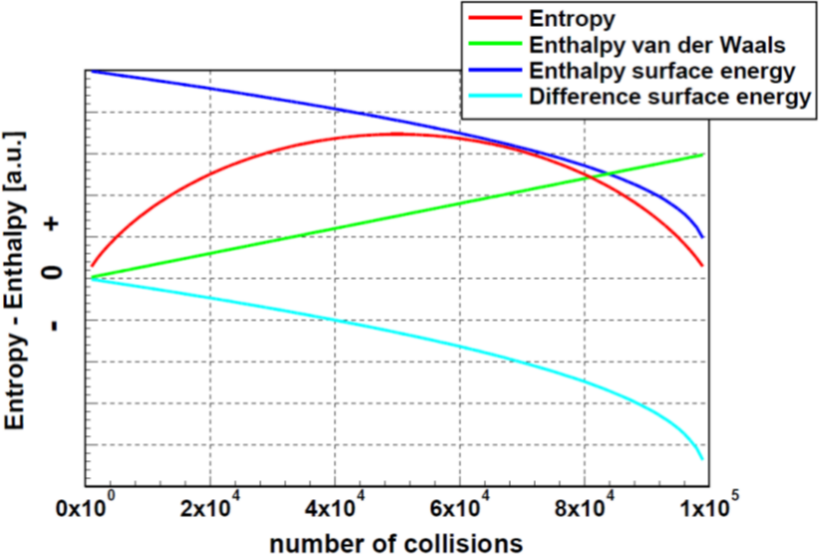
Entropy and enthalpy of agglomerated ensembles as function of the number of collisions. This simulation was calculated for an ensemble of 10^5^ particles. The enthalpies were calculated for the van der Waals interaction and the difference of the surface energy, according to Equation 5, assuming a complete agglomeration. It is important to realize that, in contrast to the entropy, the enthalpies do not show any local extremum. For clarity, the data for entropy and enthalpy are plotted in arbitrary units. Due to the large number of particles, fluctuations are, similar to Figure 4a, not visible.

As already mentioned in the section “Basic considerations”, particle agglomeration is possibly controlled by the exchange of binding energy. Certainly, also the role of the surface energy due to the reduction of the surface is relevant. Therefore, simulations were performed using Equation 5 taking care of thermal fluctuations. Thus, the agglomeration was not restricted to a reduction of the free enthalpy. As the rules for the agglomerations were, in comparison to the entropy case, not altered, the distribution function of the particle sizes is identical to the one obtained in the entropy case. The calculations led to changes of the enthalpy as function of the number of collisions as displayed in Figure 6. For the enthalpy, two assumptions were made, that is, a surface energy according to Equation 5 and van der Waals interaction. For the agglomeration ruled by the surface energy, the formation of new completely agglomerated particles was assumed. In the case of van der Waals interaction, the approach given by Luo et al. [12] was applied. Since there is nothing known about the local arrangement and interaction of the particles within an agglomerate, only one interaction per particle was assumed. This is the smallest possible number of contacts. For comparison, the entropy is plotted, too. The enthalpies, depicted in Figure 6, do not show local extrema. The data for entropy and enthalpy are plotted in arbitrary units. Certainly, also the enthalpy shows fluctuations. However, because of the large number of particles, although fluctuations can be expected, they are not visible in this graph.

### Application to a realistic example

**Figure 7:**
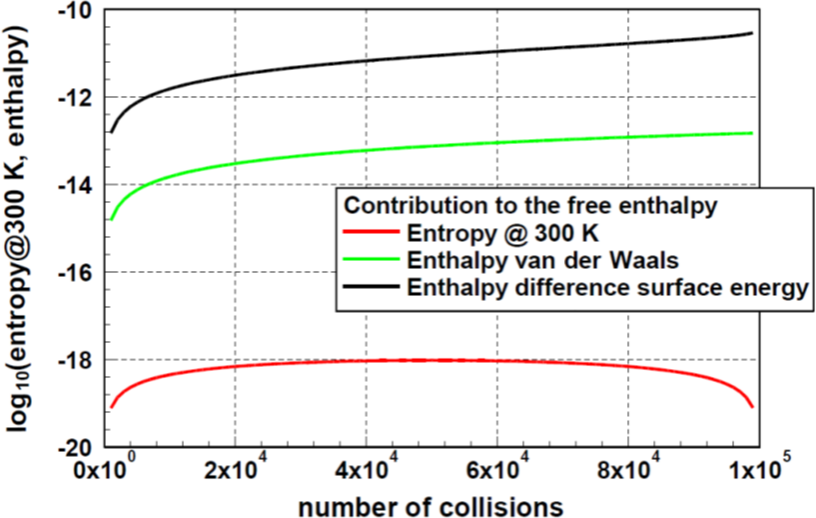
Comparison of the different possible contributions to the free enthalpy as function of the number of collisions for an ensemble of 10^5^ particles. Assuming a temperature of 300 K, one realizes that the contribution of the entropy is by far the smallest. The surface-related enthalpy was calculated assuming a particle size of 10 nm and the formation of complete new particles. As this difference is negative, for this plot the absolute value was used. Even, if one assumes only 1% of the surface as newly coagulated, this contribution remains by far the largest one.

A valid comparison of the different contributions to the free enthalpy needs a realistic example. As it offers the best availability of data, gold was selected to demonstrate the contributions of the different approaches. Basis for these calculations was the assumption of an ensemble of 10^5^ particles with a diameter of 10 nm. For the surface energy a value of 1.7 J·m^−2^ [14] was selected. The contribution of the surface energy to the enthalpy was based on the assumption that the agglomerated particles show a closed surface. Using Equation 4, the difference of the surface energy between the original and the agglomerated ensemble was calculated. For the case of van der Waals interaction the selection of the parameters is difficult. As basis, the results of Luo et al. [12] obtained for gold was applied. From these data a value of *h_i_* = 10 eV (1.6 × 10^−18^ J) was derived. Figure 7 displays the results of these calculations. Assuming a temperature of 300 K, for comparison, the contribution of the entropy is plotted as kTS*. This figure demonstrates there are a few orders of magnitude between the different contributions to the free enthalpy. As explained above, these values were calculated using extreme assumptions. For instance, let us assume that only 1% of the particles coagulate in such a way that the particles have a new common surface. Even in this case the contribution of the change in surface energy is some orders of magnitude larger than the entropy-related contribution. Similar considerations are valid in the case of van der Waals interaction.

## Discussion

The agglomeration of particles may be controlled by a few different attributes or quantities: (1) the geometry of the particles and agglomerates, (2) the minimum of the free enthalpy, and (3) the maximum of the entropy. A useful tool to analyze the above mentioned mechanisms is a simulation via MonteCarlo methods. For each step of these calculations, the agglomeration of two arbitrarily selected particles at a time is assumed. The number of particles or agglomerates in the system is reduced in this procedure by each step, although the number of particles in the system remains constant. This rule was also used as constraint for the Lagrange formalism. The influence of the geometry, the size, and of possible electrical charges of the particles was analyzed in a former paper [1]. Within the current study, the influence of thermodynamics was discussed by systematic analyses of the different thermodynamic functions.

Looking at the maximum of the entropy, the simulations show that the particle size distribution follows perfectly an exponential function. The parameters found for this exponential function are identical to those obtained from an extrema study applying the concept of Lagrange multipliers. The maximum of the entropy is always found if the number of collisions of the particles is close to half of the number of particles in the ensemble. It is important to recognize that the approach to the maximum of the entropy is influenced by fluctuations of the entropy. The next criterion analyzed was the minimum of the free enthalpy. In this context, several assumptions about the interaction were necessary. The analysis was performed either for agglomeration processes controlled by the surface energy or for processes controlled by van der Waals interaction of the particles. In case of exchange of surface energy, a complete agglomeration was assumed, in case of van der Waals interaction, one binding per particle was assumed. For comparison, the contribution of the entropy to the free enthalpy at 300 K was calculated. This finding remains correct assuming only 1% or less of the calculated enthalpy values. Concluding, one can argue that, in case of energetic interaction of the particles, the contribution of the entropy to the free enthalpy is negligibly small. This leads to the conclusion that in these cases, the criterion of maximal entropy is not applicable for the description of the agglomeration of particles.
